# Molecular Mechanisms Underlying the *In Vitro* Anti-Inflammatory Effects of a Flavonoid-Rich Ethanol Extract from Chinese Propolis (Poplar Type)

**DOI:** 10.1155/2013/127672

**Published:** 2013-01-15

**Authors:** Kai Wang, Shun Ping, Shuai Huang, Lin Hu, Hongzhuan Xuan, Cuiping Zhang, Fuliang Hu

**Affiliations:** ^1^College of Animal Sciences, Zhejiang University, Hangzhou 310058, China; ^2^Life Sciences Institute, Zhejiang University, Hangzhou 310058, China; ^3^School of Life Science, Liaocheng University, Liaocheng 252059, China

## Abstract

China produces the greatest amount of propolis but there is still lack of basic studies on its pharmacological mechanisms. Our previous study found that ethanol extract from Chinese propolis (EECP) exerted excellent anti-inflammatory effects *in vivo* but mechanisms of action were elusive. To further clarify the possible mechanisms underlying the anti-inflammatory effects of Chinese propolis (poplar type), we utilized EECP to analyze its chemical composition and evaluated its potential anti-inflammatory effects *in vitro*. High-performance liquid chromatography (HPLC) profile indicated that EECP contained abundant flavonoids, including rutin, myricetin, quercetin, kaempferol, apigenin, pinocembrin, chrysin, and galangin. Next we found that EECP could significantly inhibit the production of NO, IL-1**β**, and IL-6 in lipopolysaccharide- (LPS-) stimulated RAW 264.7 cells and suppress mRNA expression of iNOS, IL-1**β**, and IL-6 in a time- and dose-dependent manner. Furthermore, we found that EECP could suppress the phosphorylation of I**κ**B**α** and AP-1 but did not affect I**κ**B**α**'s degradation. In addition, using a reporter assay, we found that EECP could block the activation of NF-**κ**B in TNF-**α**-stimulated HEK 293T cells. Our findings give new insights for understanding the mechanisms involved in the anti-inflammatory effects by Chinese propolis and provide additional references for using propolis in alternative and complementary therapies.

## 1. Introduction

Inflammation is an integral physiological host response to tissue injury and infection, which is vital for our body when facing invading microbes or the diseases. Several relatively abundant proinflammatory mediators and cytokines are generally characterized and have been validated their important roles in inflammatory responses, such as nitric oxide (NO), interleukin (IL-)*β*, and IL-6 [1]. The overproduction of these proinflammatory mediators are typically linked to proinflammatory stimuli which will cause acute or chronic inflammatory responses and being the pathogenesis of many diseases. Increasing evidence indicates that the transcription factor nuclear factor kappa B (NF-*κ*B) as well as the activator protein (AP-)1 plays important roles in the immune/inflammatory responses by regulating the transcriptional activation of many inflammatory-related genes. In normal conditions, the inhibitor of *κ*B binding protein (I*κ*B), such as I*κ*B*α*, interacts with p50/p65 heterodimer in the cytoplasm and masks nuclear localization sequence, so the NF-*κ*B dimers failed to bind to *κ*B sites. In inflammatory conditions, I*κ*B*α* will be phosphorylated then degraded by 26 s proteosome. This leads to the free NF-*κ*B to translocate into the nucleus, binding specifically to the *κ*B sites in the genome. AP-1 is a family of DNA binding transcription factors composed of dimers with two proteins. The best-characterized AP-1 is composed by two proteins, namely Jun and Fos [2]. Various genes encoding for proinflammatory mediators, cytokines, chemokines, and some inducible enzymes contain *κ*B sites and AP-1 binding sites. The transcription of these genes is regulated, in a sense, by the NF-*κ*B and AP-1 activity [3, 4]. Therefore, suppressing or inhibiting the improper activation of these inflammation-linked transcription factors may have therapeutic potential.

Propolis is a resinous substance collected by honeybees, *Apis mellifera*, from various plant sources [5]. Bees use propolis as a building and insulating material to protect their hives. Propolis has been used in folk medicines and complementary therapies since 3,000 BC in Egypt and has become one of the most popular functional foods all around the world. Propolis show a broad spectrum of bioactivities, such as antioxidant [6], antimicrobial [7], antitumor [8], antiviral [9], anti-ulcer [10], immunomodulatory [11], cardioprotective [12], and anti-inflammatory effects [13].

Moreover, China produces the greatest amount of propolis and has become one of the largest raw propolis exporters in the world. Comparing with propolis collected from other areas, Chinese propolis also exerted lots of beneficial properties. By contrast, very limited studies focus on its pharmacological activities and functional mechanisms [14]. Our previous study found that Chinese propolis exhibited significant anti-inflammatory effects using different animal models [15]. We also demonstrated that these effects may probably attribute to the suppression of phosphatidylcholine-specific phospholipase C (PC-PLC) and to the depression of p53 and ROS levels [16]. However, detailed molecular mechanisms of the anti-inflammatory effects by Chinese propolis have not been satisfactorily elucidated.

In the present study, as a continuing part of our research to clarify the possible mechanisms underlying the anti-inflammatory effects of Chinese propolis (poplar type), we studied the total flavonoids content and analyzed the chemical composition of an ethanol extract from Chinese propolis (EECP). Furthermore, we examined effects of EECP on the *in vitro* inflammatory responses and inflammation-related transcription factors in lipopolysaccharide (LPS)-stimulated RAW 264.7 cells. We also evaluated effects of EECP on the activation of NF-*κ*B in tumor necrosis factor (TNF)-*α* stimulated HEK 293T cells. To our knowledge, this is the first time using Chinese propolis to evaluate the *in-vitro* anti-inflammatory effects in activated macrophages and effects of Chinese propolis on NF-*κ*B activation is highlighted.

## 2. Materials and Methods

### 2.1. Chemicals and Reagents

LPS (*Escherichia coli* 0127:B8) and alkaline phosphatase-conjugated secondary antibody (anti-rabbit IgG) and the standards used in HPLC analysis were purchased from Sigma-Aldrich (St Louis, USA). Primary rabbit monoclonal antibodies against phospho-c-Jun (pS63), phospho-I*κ*B*α* (pS36), and *β*-tubulin were purchased from Epitomics (Burlingame, CA, USA). The polyclonal anti-rabbit I*κ*B*α* antibody was a generous gift from Professor Zongping Xia (Life Sciences Institute, Zhejiang University, China). Other chemicals were purchased from Sangon Biotechnology Co. Ltd. (Shanghai, China).

### 2.2. Preparation of Ethanol Extract from Raw Chinese Propolis

Raw Chinese propolis sample was collected from *A. mellifera* colonies in Shandong province which is located in North China at the summer of 2010 and stored at −20°C until used. Main plant origin of the propolis sample collected was poplar (*Populus* sp.). For the subsequent experiments, one hundred grams of propolis sample was weighed and broken into powders with a grinder. Then the propolis sample was extracted by 95% (v/v) ethanol (1L), sonicated at 40°C for 3 h. After the sonication, the supernatant was filtered with Whatman No. 4 filter papers to remove the residues. The residues were collected then extracted and sonicated with 95% ethanol for another 3 h. The raw propolis was extracted for three times. Thereafter, all of the supernatants were collected together and evaporated in a rotary evaporator under a reduced pressure at 50°C. Finally, the extract was dried in the oven until reaching a constant weight and stored at −20°C until further used. During the cell experiments, the final extract was weighed and redissolved in 100% ethanol. Ethanol extract of Chinese propolis (EECP) solution was filtered with 0.22 *μ*m syringe filters (Pall, USA) to make 20 mg/mL stock and stored at −20°C in the dark. Final concentration of ethanol in the cell culture medium did not exceed than 0.1% (v/v).

### 2.3. Total Flavonoids Measurement and HPLC Analysis

Total flavonoids content of propolis extract was measured using the method of Chinese Standard (GB/T 20574-2006). The absorbance reading was determined by an Ultraviolet Specterphotometry (UV-2550, SHIMADZU Co., Japan) at 415 nm. The chromatographic analyses were carried out using the method of Chinese Standard (GB/T 19427-2003) and operated on an Agilent HPLC system equipped with a vacuum degasser G1322A, a quaternary pump G1311A, an autosampler G1329A, a programmable variable wavelength detector (VWD) G1314B, and a thermostated column compartment G1316A. Separation of flavonoids was performed on Sepax GP-C18 column (4.6 mm × 150 mm, 3 *μ*m) at 28°C with a detection wavelength at 270 nm. The mobile phases were consisted of methanol and water at a ratio of 58/42 (v/v) and the pH value was adjusted to 3 with phosphoric acid at a flow rate of 0.7 mL/min. All of the sample solutions were filtered through 0.22° *μ*m membrane filters with an injection volume of 10 *μ*L. 

### 2.4. Cell Culture and Cell Viability Evaluation by MTT Assay

Murine macrophages-like cell line RAW 264.7 and human embryonic kidney (HEK) 293T cells originated from the American Type Culture Collection (ATCC) and cultured in DMEM (high glucose) supplemented with 100 U/mL of penicillin, 100 *μ*g/mL streptomycin and 10% heat-inactivated fetal bovine serum (Gibco, USA) at 37°C and 5% CO_2_ in a humidified incubator. Effect of EECP on the viability of RAW 264.7 cells was determined by 3-(4,5-dimethylthiazol-2-yl)-2,5-diphenyltetrazolium bromide (MTT) assay as previously described [17] using the MTT Cell Proliferation and Cytotoxicity Assay Kit (Beyotime, Beijing, China) according to manufacturer's instructions. The optical density values were measured at 570 nm using an ELISA reader (Bio-Rad 680, USA).

### 2.5. NO Measurement by Greiss' Reaction and Cytokine Determination by ELISA

Murine RAW 264.7 cells (1.2 × 10^5^) were seeded into 24-well plates and cultured for 24 h, thereafter the cells were pretreated with specified concentrations of EECP for 1 h then the cells were stimulated with 1 *μ*g/mL LPS. After incubating at designated length of time, the cell medium was collected and centrifuged, then dispensed and stored at −80°C until tested. The amount of the inflammatory-related cytokines, IL-1*β* and IL-6 in the cell culture supernatants was measured using enzyme-linked immunosorbent assay (ELISA) kits, according to manufacturer's instructions (Sunny ELISA Kits, Mutisciences, Hangzhou, China). The generation of nitric oxide was assayed using a NO measurement kit by a colorimetric Griess' method (Beyotime, Beijing, China). The optical density was measured at 450 nm for IL-1*β* and IL-6 and at 550 nm for NO using the ELISA reader (Bio-Rad 680, USA).

### 2.6. Total RNA Isolation and Reverse Transcription

Total RNA was isolated from RAW 264.7 cells using an E.Z.N.A total RNA Kit (Omega Bio-tech Inc., USA) following the protocol provided by the manufacturer. The purity and concentration of all RNA samples were measured using a Nano Drop spectrophotometer (ND-2000, NanoDrop Technologies, USA). Total RNA samples were suspended in DEPC-treated water and stored at −80°C until further used. For cDNA synthesis, 1 *μ*g of total RNA were used in a 25 *μ*L reaction volume using a First-Strand cDNA synthesis kit (GeneCopoeia, USA). The reaction products of reverse transcription were kept frozen at −20°C until used.

### 2.7. Quantitative Real-Time Polymerase Chain Reaction Analysis

All of the oligonucleotide primers used were designed using the Perlprimer software and synthesized commercially (Sangon Biotechnology, Shanghai, China). The sequences of the primers were as follows: murine GAPDH sense, 5′-GAGAAACCTGCCAAGTATGATGAC-3′, GAPDH anti-sense, 5′-TAGCCGTATTCATTGTCATACCAG-3′; IL-1*β* sense, 5′-CCAACAAGTGATATTCTCCATGAG-3′, IL-1*β* anti-sense, 5′-ACTCTGCAGACTCAAACTCCA-3′; IL-6 sense, 5′-CTCTGCAAGAGACTTCCATCC-3′, IL-6 anti-sense, 5′-GAATTGCCATTGCACAACTC-3′. iNOS sense, 5′-TTTCCAGAAGCAGAATGTGACC-3′, iNOS anti-sense, 5′-AACACCACTTTCACCAAGACTC-3′. Suitable sizes of synthesized cDNA were 239 bp for IL-1*β*, 210 bp for IL-6, 294 bp for iNOS, and 212 bp for GAPDH. Quantitative real-time PCR was performed with the Mastercycler ep realplex (Eppendorf, Hamburg, Germany) using a SYBR premix EX Taq (TaKaRa, Dalian, China) following manufacturer's protocols. The reactions were carried out in duplicate in a 25 *μ*L reaction volume in a 96-well plate format and the reaction mixtures with no cDNA served as negative control. The 2-steps PCR reaction condition was as follows: initial denaturation at 95°C for 30 s, followed by 40 cycles of denaturation at 95°C for 5 s, annealing and extension at 60°C for 30 s, followed by the confirmation with the melting curve analysis at 95°C for 15 s, 50°C and 95°C for 15 s. The real-time PCR products were also confirmed by DNA sequencing and electrophoresed with 1.5% agarose gel after staining by GoldView (SBS Genetech, Beijing, China), visualized under UV light. GAPDH was used as a housekeeping gene to normalize the expression of the target genes (IL-1*β*, IL-6, and iNOS) using a  ^ΔΔ^Ct method [18]. 

### 2.8. Cellular Protein Extraction and Western Blotting Analysis

RAW 264.7 cells were pretreated with assigned concentrations of EECP for 1 h. Then the cells were stimulated with 1 *μ*g/mL LPS at pre-designed time points. At the harvest time, the cells were put on the ice immediately and washed twice with cold PBS. Then the cells were lysed on ice for 10 min using a cell lysis buffer containing 50 mM Tris-Cl (pH 7.5), 150 mM NaCl, 0.5% NP-40, 10% glycerol, 2 mM DTT, 1 mM leupeptin, and 1 mM PMSF. All the lysate were collected by scraping with the cell scrapers (Corning, USA) and centrifugated at 4°C at a speed of 12,000 g for 10 min to remove cell debris. After that, equal amounts of cellular protein (30 *μ*g) were mixed with a quarter-volume of the Laemmli's sample buffer and boiled at 95°C for 5 min. Then the cellular proteins were separated by 12–15% sodium dodecyl sulfate-polyacrylamide gel electrophoresis (SDS-PAGE). Afterwards, the gels were transferred to polyvinylidene fluoride (PVDF) membranes and 5% skim milk dissolved in Tris-buffered saline Tween 20 (TBST, 20 mM Tris-Cl, pH 7.4, 150 mM NaCl, and 0.02% Tween 20) was used to block the nonspecific binding sites on the PVDF membrane (Millipore, USA) for 30 min at room temperature. Then the blots were incubated with primary antibodies for 1 h at room temperature or 4°C overnight. Thereafter, the membranes were washed for three times with TBST and incubated with a 1 : 10,000 dilution of alkaline phosphatase-conjugated secondary antibody for 1 h at room temperature. After another three times washing with TBST, the immunoreactive protein bands on the membrane were developed for 3 min in 10 mL alkaline phosphatase for western color development buffer (100 mM Tris-Cl, pH 9.5, 50 mM NaCl and 5 mM MgCl_2_) mixed with 100 *μ*L NBT/BCIP solution (18.75 mg/mL Nitro blue tetrazolium chloride, NBT and 9.4 mg/mL 5-bromo-4-chloro-3-indolyl phosphate toluidine salt, BCIP in 67% DMSO, v/v). The Western blotting results were evaluated using Quantity one software if necessary.

### 2.9. Transient Transfection and NF-*κ*B Reporter Assay

In order to examine the effect of EECP on NF-*κ*B activation, HEK 293T cells were plated in 12-well plates at 1.5 × 10^5^ cells per well and incubated overnight. Cells were transfected the next day with 30 ng of firefly luciferase reporter plasmid (pGL4.2-3 × NF-*κ*B-Luc) and 5 ng of sea pansy luciferase reporter plasmid (pRL-TK). Total expression plasmids are 500 ng and the pcDNA3.1 vector was added as needed to make up the total amount of expression plasmid DNA. 24 h after transfection, cells were pretreated with various concentrations of EECP solutions (from 0 to 20 *μ*g/mL) for 1 h, then stimulated with 10 ng/mL of TNF-*α* for 12 h. Cell viability was confirmed by trypan blue exclusion and microscopy examination. Then the cells were washed with cold PBS twice and harvested in the cell lysis buffer (0.5% CHAPS, 25 mM glycylglycine, 15 mM MgSO_4_ and 4 mM EGTA, 1 mM DTT and PMSF). After that, the cell lysate were kept on the shaker at room temperature for 20 min, then transferred to the ice. Afterwards, move the lysate to Eppendorf tubes and spinned for 10 minutes at 12,000 g. Firefly and sea pansy luciferase activities were quantified using a DLReady luminometer (Berthold Technologies, Germany), with firefly luciferase activity normalized for transfection efficiency based on sea pansy luciferase activity.

### 2.10. Statistical Analysis

All data are representative of at least three independent experiments. Data are presented as mean ± SD and student's *t*-test was used to demine the statistical significance between two groups and *P* value of <0.05 was considered to be statistically significant.

## 3. Results

### 3.1. Total Flavonoids Content of EECP and Phytochemical Analysis by HPLC

**Figure 1:**
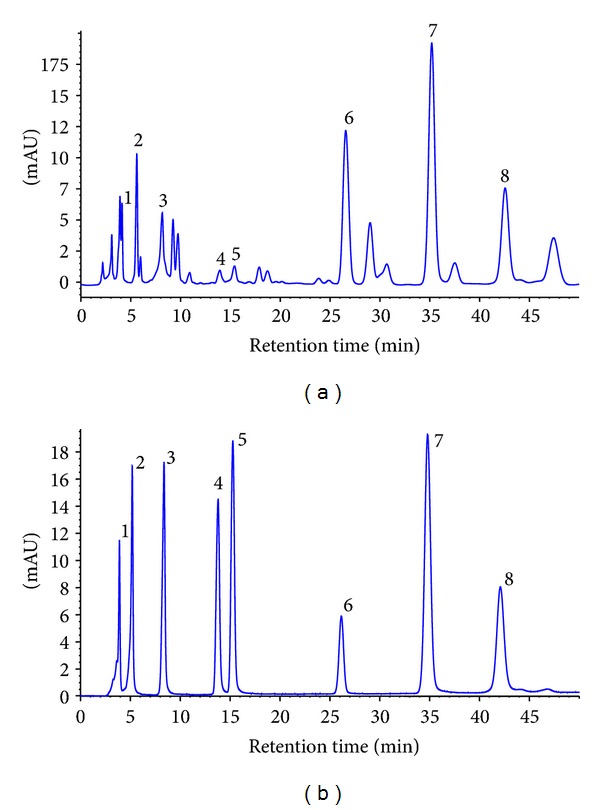
HPLC chromatogram of (a) EECP and (b) flavonoids standards. Peaks: (1) rutin; (2) myricetin; (3) quercetin; (4) kaempferol; (5) apigenin; (6) pinocembrin; (7) chrysin; and (8) galangin.

The chromatographic profile of EECP and the flavonoid standards were recorded at 270 nm in Figure 1 and their amounts are shown in Table 1. Total flavonoids content of EECP was 227.24 ± 1.01 mg rutin equivalent per gram using a colorimetric method. We also analyzed eight most representative flavonoids of poplar tree type propolis, namely rutin, myricetin, quercetin, kaempferol, apigenin, pinocembrin, chrysin, and galangin. The HPLC file showed that all of the eight flavonoids are presented in EECP, in accordance with previous study [19].

### 3.2. Assessment of Cell Toxicity of EECP in RAW 264.7 Cells

**Figure 2:**
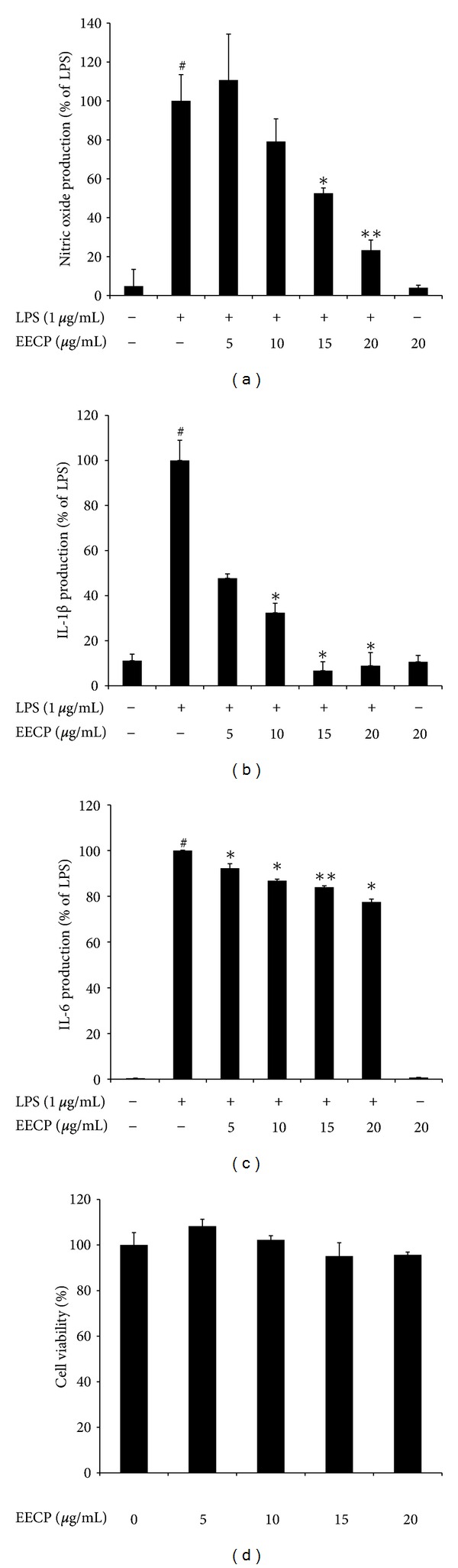
Effects of EECP on LPS-induced NO, IL-1*β*, and IL-6 production and cell viability in RAW 264.7 cells. (a–c) Cells were pretreated with/without indicated concentrations of EECP for 1 h then stimulated with LPS (1 *μ*g/mL) for 18 h. Control values were obtained in the absence of LPS or EECP. The values are presented as percentages of NO (a), IL-1*β* (b), and IL-6 (c) comparing with LPS-treated cells, respectively. (d) RAW 264.7 cells were treated with indicated concentrations of EECP (0–20 *μ*g/mL) for 24 h, and the results are expressed by percentages of surviving cells over control cells (no addition of EECP) using MTT assays. The data are the means ± SDs for three independent experiments. Individual groups were compared using Student's *t*-test (*P < 0.05, **P < 0.01 compared with the LPS group; ^#^ *P* < 0.05 compared with untreated group).

Cytotoxicity of EECP on RAW 264.7 cells is shown in Figure 2(d). After incubating for 24 h, EECP at concentrations up to 20 *μ*g/mL had no significant effects on the cell viability (*P* > 0.05). However, concentrations higher than that amount will be toxic to RAW 264.7 cells (data not shown). With this result, we chose concentrations of EECP up to 20 *μ*g/mL in the subsequent experiments.

### 3.3. Effects of EECP on the Production of NO, IL-1*β* and IL-6 in LPS-Stimulated RAW 264.7 Cells

Figures 2(a), 2(b), and 2(c) show the effects of EECP on NO, IL-1*β* and IL-6 production in LPS-stimulated RAW 264.7 cells. Our previous data found Chinese propolis exhibited good anti-inflammatory effects *in vivo* and EECP could inhibit the production of some inflammation-related cytokines [12]. So we first investigated effects of EECP on the production of some proinflammatory cytokines, including IL-1*β*, and IL-6 as well as the NO generation in LPS-stimulated RAW 264.7 cells. ELISA assays were used to determinate the proinflammatory cytokines and Greiss' reaction was used to estimate the NO generation. In the absence of LPS, very low amounts of NO and those two proinflammatory cytokines were detected in the culture supernatants of RAW 264.7 cells. Upon stimulation with LPS (1 *μ*g/mL), NO production was markedly increased (Figure 2(a)). We also found significant increases of IL-1*β* and IL-6 in the presence of LPS-stimulation (Figures 2(b) and 2(c)). However, pretreatment with EECP for 1 h could significantly reduce the production of NO, IL-1*β* and IL-6 in a dose-dependent manner (Figures 2(a) to 2(c)).

### 3.4. Effects of EECP on iNOS, IL-1*β* and IL-6 mRNA Expression in LPS-Stimulated RAW 264.7 Cells

**Figure 3:**
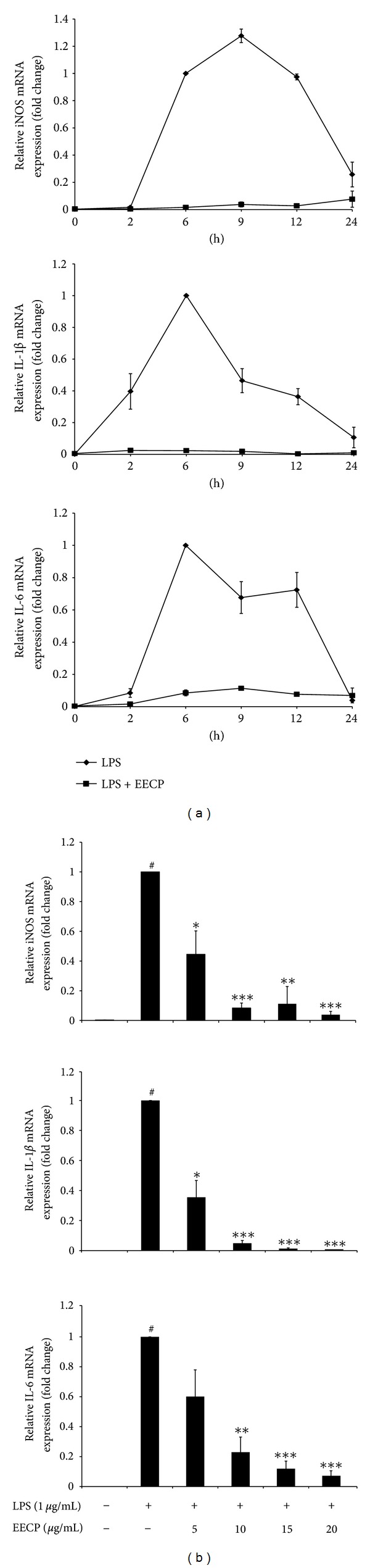
Effects of EECP on iNOS, IL-1*β* and IL-6 mRNA expression in LPS-stimulated RAW 264.7 cells. Dose-dependent (a) and time-course (b) inhibitory effect of EECP were measured on LPS-induced iNOS IL-1*β* and IL-6 mRNA expression in RAW 264.7 cells were measured using qRT-PCR, as described in Section 2. (a) Cells were pretreated with EECP (15 *μ*g/mL) or not for 1 h, then stimulated with LPS (1 *μ*g/mL) at various time points. (b) Dose-effect relationship was measured after 6 h stimulation with LPS (1 *μ*g/mL). Results are expressed as a target gene expressions ratio comparing with the LPS-treated group. The data are the means ± SDs for three independent experiments. Individual groups were compared using student's *t*-test (*P < 0.05, **P < 0.01, ***P < 0.001 compared with the LPS group; ^#^ *P* < 0.05 compared with untreated group).

Figure 3 shows the effects of EECP on mRNA expression of iNOS, IL-1*β*, and IL-6 in LPS-stimulated RAW 264.7 cells. Time- and dose-effect of EECP on the mRNA expression of iNOS, IL-1*β*, and IL-6 was measured in RAW 264.7 cells stimulated with LPS in the presence or absence of EECP using quantitative real-time reverse transcriptase polymerase chain reaction (qRT-PCR). As expected from several previous studies, upon stimulation with LPS, mRNA expressions of three inflammatory-related genes in RAW 264.7 cells were upregulated strongly [20, 21]. The mRNA expression reached a peak level at about 6 h for IL-1*β* and IL-6, 9 h for iNOS. Pretreatment of EECP at 15 *μ*g/mL could block the expression of these mRNAs at all of indicated time points (Figure 3(a)). We also observed that pretreatment of various concentrations of EECP on the inhibition of LPS-induced mRNA levels of those three genes is dose-dependent (Figure 3(b)). 

### 3.5. Effects of EECP on the Degradation and Phosphorylation of I*κ*B-*α* in LPS-Stimulated RAW 264.7 Cells

**Figure 4:**
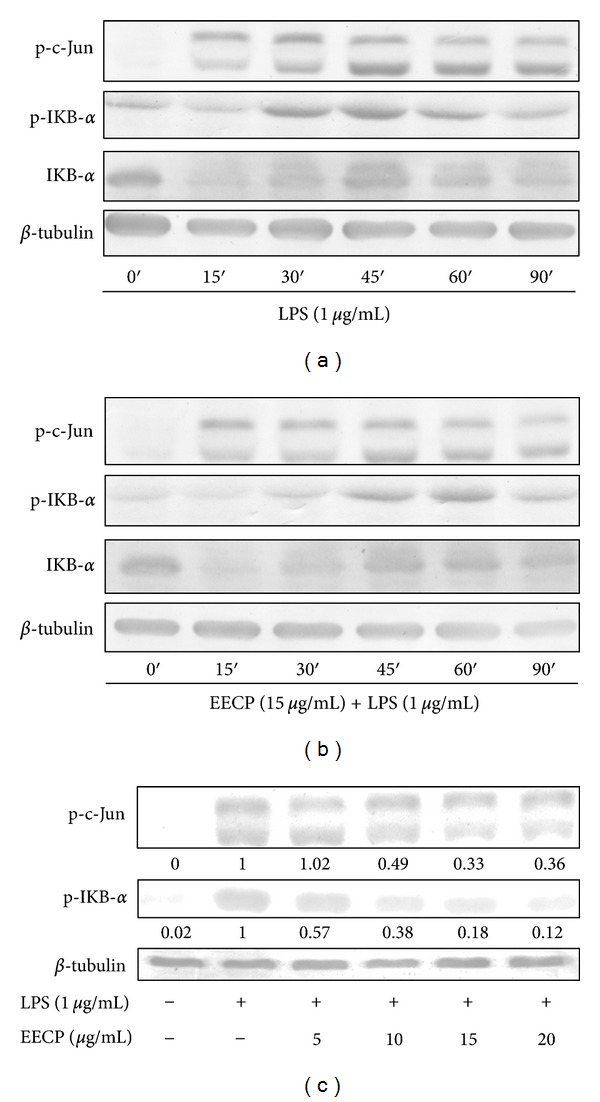
Effects of EECP on the phosphorylation of I*κ*B-*α* and AP-1 in LPS-stimulated RAW 264.7 cells. (a) RAW 264.7 cells were stimulated with LPS (1 *μ*g/mL) for indicated times or (b) were pretreated with EECP (15 *μ*g/mL) for 1 h then stimulated with LPS (1 *μ*g/mL) for indicated times. (c) RAW 264.7 cells were pretreated or not with indicated concentrations of EECP for 1 h then were stimulated with LPS (1 *μ*g/mL) for 30 min. Whole cell lysates were analyzed by Western blotting analysis using specific antibodies. The relative expression of proteins was quantified using Quantity One software comparing with *β*-tubulin. Data shown are the representative of three independent experiments with similar results.

Figure 4 indicates total and phosphorylation protein expression levels of I*κ*B-*α* in the cytoplasm. In order to further clarify the molecular mechanisms underlying EECP-mediated suppressive effects of those inflammation-related genes, we next used Western blot analysis with specific antibodies to characterize effects of EECP on LPS-induced I*κ*B*α* phosphorylation and degradation, which were regarded as two symbolic events in the activation of NF-*κ*B signal transduction. A time-course experiment showed that the phosphorylation of I*κ*B*α* was increased from 15 min after LPS treatment and application of 15 *μ*g/mL EECP attenuated LPS-induced phosphorylation of I*κ*B*α*. In contrast, comparing with LPS treated cells, the degradation of I*κ*B*α* seemed was unaffected by combination of EECP and LPS (Figures 4(a) and 4(b)). A dose-course experiment showed EECP at a concentration of 10 to 20 *μ*g/mL could block the LPS-induced phosphorylation of I*κ*B*α* (Figure 4(b)) but has no significant effect on preventing the degradation of I*κ*B*α* (data not shown). 

### 3.6. Effect of EECP on the Phosphorylation of AP-1 in LPS-Stimulated RAW 264.7 Cells

Figure 4 also shows the phosphorylation levels of c-Jun, a major component of AP-1 in LPS-stimulated RAW 264.7 cells. Previous studies have found that many inflammatory mediators, such as iNOS, IL-1*β* and IL-6 were regulated not only by NF-*κ*B but also by AP-1, another important transcription factor involved in MAPK signaling pathways. Following LPS stimulation, the AP-1 heterodimer, c-Jun and c-Fos translocated into the nucleus, leading to the transcription of several inflammatory-related genes [4]. To further explore whether AP-1 is involved in the inhibition of those inflammatory factors by EECP, we examined time- and dose-effect of EECP on the phosphorylation of c-Jun in LPS-stimulated RAW 264.7 cells. After LPS stimulation, the phosphorylation level of c-Jun was dramatically increased from 15 min and at a peak level at about 30 min (Figure 4(a)). Pretreatment with 15 *μ*g/mL of EECP could postpone c-Jun's phosphorylation at the peak level at about 60 to 90 min (Figure 4(b)). EECP could also suppress LPS-induced phosphorylation of c-Jun (AP-1) in a dose-dependent manner (Figure 4(c)). 

### 3.7. Effect of EECP on NF-*κ*B Activation in TNF-*α* Treated HEK 293T Cells

**Figure 5:**
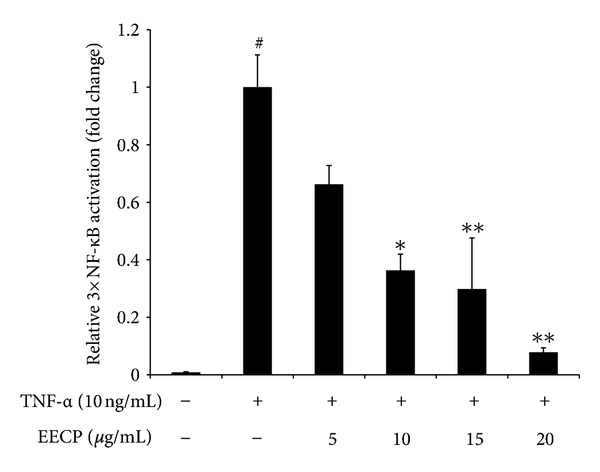
Effect of EECP on NF-*κ*B activation in TNF-*α* treated HEK 293T cells. HEK 293T cells were transiently transfected with firefly luciferase reporter plasmid (pGL4.2-3×NF-KB-Luc) and luciferase reporter plasmid (pRL-TK) for 24 h. Then cells were pretreated with an increasing concentrations of EECP (0–20 *μ*g/mL) in the presence of TNF-*α* (10 ng/mL). Unstimulated cells were used as a negative control. Luciferase activity was normalized to tk-Renilla luciferase activity and results are expressed by the percentage of TNF-*α* treated group. Data represent the mean ± SD of three independent experiments. Individual groups were compared using student's *t*-test (*P < 0.05, **P < 0.01, ***P < 0.001 compared with the LPS group; ^#^ *P* < 0.05 compared with untreated group).

Figure 5 shows effect of EECP on NF-*κ*B activation in TNF-*α* treated HEK 293T cells. Paulino et al. previously demonstrated that ethanol extracts of Brazilian green propolis exerted a strong suppressive effect on NF-*κ*B activation using a reporter assay system with an IC_50_ value of 200 *μ*g/mL [13]. However, botanical origins and chemical compositions between Brazilian green propolis and Chinese propolis are quite different, so we evaluated the effect of EECP on the NF-*κ*B activation using a reporter assay system in TNF-*α* treated HEK 293T cells. As shown in Figure 5, the up-regulation of the relative luciferase activity stimulated by TNF-*α* (10 ng/mL) was significantly (*P* < 0.05) decreased by EECP (10 to 20 *μ*g/mL).

## 4. Discussion

The present study was undertaken to elucidate the mechanisms underlying the anti-inflammatory and immunomodulating effects of a flavonoid-rich ethanol extract from Chinese propolis (poplar type), which has been widely used in folk medicine and health food among China and all over the world [5]. Indeed, chemical constituents of propolis vary widely with respect to their geographic regions and plant sources. However, biological activities of propolis always present. As far as Chinese propolis, the raw material we used in this study is origin from Shandong province which is located in North China and the main botanical source of this type of propolis is well known as poplar (*Populus sp.*) [22]. Based on previous studies, we first chose eight most frequently-presented flavonoids in poplar type propolis, including rutin, myricetin, quercetin, kaempferol, apigenin, pinocembrin, chrysin, and galangin to analyzed their relative amount in EECP [14, 23]. The chromatographic profile of EECP meets the characters of the poplar-type propolis, which is marked by high amounts of flavonoids (including flavones, flavonols, flavanones and dihydroflavonols) and other phenolics (mainly cinnamic acids and their esters) [24]. 

It is known that macrophage plays an important role in the immune system as well as the inflammation process. LPS-stimulated macrophages can secrete a variety of inflammatory mediators, including NO, IL-1*β*, and IL-6. Overproduction of these mediators will break the immune homeostasis and cause several inflammatory diseases, including septic shock, arteriosclerosis, and cancer. To continue our work to clarify the mechanisms underlying the anti-inflammatory effects of EECP, we first chose LPS-stimulated RAW 264.7 cells as an *in vitro* model to evaluate the effects of ethanol extract from Chinese propolis (poplar type) on the production and mRNA expression of those inflammatory mediators. Our results showed that LPS-stimulation strongly up-regulated the production and secretion of NO, IL-1*β* and IL-6. NO is one ubiquitous cellular mediator, which is produced by iNOS, which can generate and modify intracellular signals. During the pathological process, overproduction of NO is harmful [25]. Parallel to previous study, we also found that EECP could significantly reduce NO production and iNOS mRNA expression in activated macrophages regardless of the geographic locations of the samples used in these experiments [26, 27]. Meanwhile, in activated macrophages, IL-1*β* and IL-6 are known as two important proinflammatory cytokines which can cause the aggravation of inflammation and induce tissue damages [28]. Previous study using LPS-activated J744A.1 macrophages model found ethanol extract of propolis could significantly suppress the production and expression of IL-1*β* in a dose-dependent manner. Recently, effects of propolis and its isolated compounds, on some cytokines production (IL-1*β*, IL-6, and IL-10) has been investigated. Bachiega et al. found that ethanol extracts of propolis (collected in south area of Brazil) could suppress the cytokines production induced by LPS both before and after its addition [29]. In the present study, we also found EECP could suppress the production of IL-1*β* and IL-6. Meanwhile, using qRT-PCR we found that EECP on the inhibition of LPS-induced mRNA levels of iNOS, IL-1*β*, and IL-6 is time- and dose-dependent, indicating that EECP exerts a suppressive effect against the inflammation-related genes at transcriptional level.

Since the transcriptional upregulation of inflammatory mediators is reported to be due to some redox sensitive transcription factors such as NF-*κ*B and AP-1 [2, 3], we hypothesize that EECP could be involved in the modulation of these transcription factors. In LPS-induced models, activated macrophages produce numerous proinflammatory mediators most of which is regulated by NF-*κ*B transcription factor and this pivotal signaling pathway mediating inflammatory responses has been well-established by many previous reports [18, 28]. Similar to previous studies, our results found that LPS-stimulation led to a rapid phosphorylation and degradation of I*κ*B*α* that peaked at 30 min. EECP alone could not induce any change of I*κ*B*α* in resting RAW 264.7 cells (data not shown), but it had a strong inhibition effect on phosphorylation of I*κ*B*α*. However, effect of EECP on preventing the degradation of I*κ*B*α* is not very obvious. Interestingly, NF-*κ*B reporter assay result showed that EECP also displayed a strong suppressive effect on the activation of NF-*κ*B in TNF-*α* activated HEK 293T cells, regardless of the geographic location and the propolis samples used [13]. Further studies should focus on the effects of some other essential upstream or downstream events associated with NF-*κ*B cascade, such as the phosphorylation of I*κ*B*α* kinase (IKK) or the translocation of NF-*κ*B. On the other hand, AP-1 is another important transcriptional factor during the transcription and production of some inflammatory mediators. Many inflammatory-related genes not only contain *κ*B sites but also has AP-1 binding sites in their promoters. However, effect of propolis extracts on the transcription of AP-1 is still elusive. Upon stimulation with 1 *μ*g/mL LPS, the phosphorylation of AP-1 reached a peak level at 30–45 min, then decreased. This result is a slight inconformity with previous study. Shan et al. found the phosphorylation of AP-1 reached a peak level after stimulation with LPS for 1 h [20]. We considered that this inconformity is acceptable, for the LPS amount we used is quite different; they used 100 ng/mL instead of 1 *μ*g/mL LPS we used. High amounts of LPS used seem promoted the phosphorylation and transcription of AP-1. Even so, pretreated with EECP before the stimulation displayed a potential suppressing effect on the phosphorylation of AP-1, gave us another explanation to the attenuation of those proinflammatory cytokines production by EECP pretreatment before the LPS stimulation.

Although numerous studies reported that propolis has a wide spectrum of pharmacological activities, we should not ignore the main constituent of propolis from different geographic locations is quite different. Various factors, such as plant resources, collecting seasons, species of bees, and the solvents used in extraction, will influence the chemical constituent of propolis [22, 24]. So it is necessary to distinguish between specific propolis types and its corresponding compositions. In contrast with other types of propolis, such as Brazilian green propolis, birch type propolis, or red propolis, the samples we used is originated from Shandong, and only can be seen as a representative of poplar type propolis of Northern China. The phytochemistry analysis also found that it contains multiple kinds of flavonoids at a relatively high amount. Among them we found that pinocembrin (84.6 ± 0.2 mg/g of the extract), chrysin (33.5 ± 0.1 mg/g of the extract) and galangin (29.1 ± 0.2 mg/g of the extract) are three of the most abundant flavonoids presented in EECP. Previous studies have confirmed their effects on activated RAW 264.7 macrophages [30]. Yu et al. found that pinocembrin could inhibit LPS-stimulated nitric oxide (NO) and prostaglandin E2 (PGE2) production in RAW 264.7 cells by blocking the activation of NF-*κ*B [31]. Similar effects were also observed using chrysin [32]. It has also reported chrysin and galangin could suppress the IL-1*β* gene expression in activated RAW 264.7 macrophages via inhibiting the gene transcription [33]. 

We also noticed that some previous reports found that propolis could activate the macrophage and induce NO and TNF-*α* production [34]. Some *in vitro* and *in vivo* assays also demonstrated that propolis can modulate the action of murine peritoneal macrophages and increase their resistance against some microbes [21, 35]. It is worth noting that the mechanisms seem to differ by the types of propolis used, the extraction method used, even the cell-line and the stimulus. Many isolated constituents presented in propolis have been found with properties of immunoregulation. For example, some constituents in propolis may exert an immunosuppressive effect, such as caffeic acid phenyl ester (CAPE), artepillin C (3,5-diprenyl-4-hydroxycinnamic acid). CAPE can inhibit the induction of cytokines, as well as iNOS and cyclooxygenase (COX)-2 expression, after *in vitro* LPS stimulation [36]. CAPE is also a potent inhibitor of T-cell-receptor-mediated T cell proliferation and often acts as an inhibitor of NF-*κ*B [37]. Paulino et al. found that artepillin C could suppress macrophage activation and block the activity of NF-*κ*B [38]. However, some other constituents presented in propolis were reported with immunostimulatory effects, including cinnamic and coumaric acids [29, 39]. So we speculate that the effects of various kinds of propolis may also attribute to their synergic effect among various compounds. Some of those constituents may act both cooperatively and antagonistically. Total effect of propolis, without any doubt, depends on the composition of those biologically active ingredients and their concentrations.

In conclusion, our study confirmed the *in-vitro* anti-inflammatory effects of a flavonoid-rich ethanol extract from Chinese propolis (poplar type). We studied the major constituents of EECP and eight flavonoids have been identified. We also observed that EECP could block the production of NO, IL-1*β*, and IL-6 in LPS-stimulated RAW 264.7 cells. EECP was able to regulate the mRNA expression of iNOS, IL-1*β*, and IL-6 in a time- and dose-dependent manner. These effects seem to be mediated, at least in part, by inhibiting the phosphorylation of I*κ*B*α* and AP-1. We also found EECP could block NF-*κ*B activation in TNF-*α* stimulated HEK 293T cells. Nevertheless, we still cannot rule out other possible mechanisms may be involved. Our study gives a new insight for understanding the mechanisms involved in anti-inflammatory effect by Chinese propolis as well as provides some references for using propolis in alternative and complementary therapies in the future.

## Figures and Tables

**Table 1:** Concentrations of flavonoids presented in EECP.

Compounds	Retention Time (min)	mg/g of extract^a^
Rutin	4.13	18.8 ± 0.1
Myricetin	5.61	15.4 ± 0.2
Quercetin	8.16	19.7 ± 0.3
Kaempferol	13.94	3.7 ± 0.1
Apigenin	15.41	3.5 ± 0.1
Pinocembrin	26.57	84.6 ± 0.2
Chrysin	35.20	33.5 ± 0.1
Galangin	42.57	29.1 ± 0.2

^a^Reported values are the means ± SD (*n* = 3). EECP, ethanol extracts of Chinese propolis.

## Abbreviations

EECP: Ethanol extract of Chinese propolis
HPLC: High performance liquid chromatography
NO: Nitric oxide
IL: Interleukin
iNOS: Inducible form of nitric oxide synthase
I*κ*B: Inhibitor of *κ*B binding protein
NF-*κ*B: Nuclear factor kappa B
AP-1: Activator protein-1
LPS: Lipopolysaccharide
TNF-*α*: Tumor necrosis factor *α*
PC-PLC: Phosphatidylcholine-specific phospholipase C
ROS: Reactive oxygen species
PGE2: Prostaglandin E2
CAPE: Caffeic acid phenyl ester.
